# IgSF11 regulates osteoclast differentiation through association with the scaffold protein PSD-95

**DOI:** 10.1038/s41413-019-0080-9

**Published:** 2020-02-10

**Authors:** Hyunsoo Kim, Noriko Takegahara, Matthew C. Walsh, Sarah A. Middleton, Jiyeon Yu, Jumpei Shirakawa, Jun Ueda, Yoshitaka Fujihara, Masahito Ikawa, Masaru Ishii, Junhyong Kim, Yongwon Choi

**Affiliations:** 1 Department of Pathology and Laboratory Medicine, University of Pennsylvania Perelman School of Medicine, Philadelphia, PA 19104 USA; 2 Department of Biology, Department of Computer and Information Science, School of Arts and Sciences, Program in Single Cell Biology, University of Pennsylvania, Philadelphia, PA 19104 USA; 3 Research Institute for Microbial Diseases, Osaka University, Suita, Osaka 565-0871 Japan; 4 Department of Immunology and Cell Biology, Graduate School of Medicine and Frontier Biosciences, Osaka University, Suita, Osaka 565-0871 Japan

**Keywords:** Bone, Bone quality and biomechanics

## Abstract

Osteoclasts are multinucleated, giant cells derived from myeloid progenitors. While receptor activator of NF-κB ligand (RANKL) stimulation is the primary driver of osteoclast differentiation, additional signaling further contributes to osteoclast maturation. Here, we demonstrate that immunoglobulin superfamily member 11 (IgSF11), whose expression increases during osteoclast differentiation, regulates osteoclast differentiation through interaction with postsynaptic density protein 95 (PSD-95), a scaffold protein with multiple protein interaction domains. IgSF11 deficiency in vivo results in impaired osteoclast differentiation and bone resorption but no observed defect in bone formation. Consequently, IgSF11-deficient mice exhibit increased bone mass. Using in vitro osteoclast culture systems, we show that IgSF11 functions through homophilic interactions. Additionally, we demonstrate that impaired osteoclast differentiation in IgSF11-deficient cells is rescued by full-length IgSF11 and that the IgSF11-PSD-95 interaction requires the 75 C-terminal amino acids of IgSF11. Our findings reveal a critical role for IgSF11 during osteoclast differentiation and suggest a role for IgSF11 in a receptor- and signal transduction molecule-containing protein complex.

## Introduction

Bone homeostasis is maintained by the balanced functions of bone-forming osteoblasts and bone-resorbing osteoclasts.^1,2^ Excessive osteoclast activity can cause pathogenic bone loss,^3^ so investigations of the molecular signaling and genetic programs that control osteoclast differentiation and function are required both to improve our understanding of osteoclast biology and to provide a molecular basis for designing therapeutic strategies for bone remodeling diseases.^4^

Osteoclasts are specialized multinucleated, giant cells derived from bone marrow precursors of the monocyte/macrophage lineage.^5,6^ Osteoclast differentiation is initiated and sustained primarily by the osteoclast differentiation factor RANKL,^7^ which is mainly produced by osteoblasts and osteocytes.^8–10^ However, osteoclast differentiation also requires costimulation by ITAM-associated surface receptors and cell–cell interactions mediated by cell adhesion molecules (CAMs).^11^ CAMs are required not only to establish cell–cell contacts but also to mediate intracellular signal transduction for optimal activation and/or differentiation of the interacting cells.^5,12,13^

Immunoglobulin superfamily member 11 (IgSF11) was originally identified as a member of the immunoglobulin superfamily,^14^ and it has been revealed to function as a CAM in a calcium-independent manner.^15,16^ IgSF11 has been shown to be predominantly expressed in the brain and testis in mammals.^14^ In the brain, IgSF11 has been demonstrated to regulate synaptic transmission and plasticity^17^ and has also been shown to be involved in the development of the mouse cerebellum by suppressing the proliferation and promoting the differentiation of cerebellar granule cell precursors into cerebellar granule cells.^18^ In the testis, IgSF11 has been demonstrated to be expressed in Sertoli cells and to maintain a functional blood–testis barrier.^19^ Recently, IgSF11 has been identified as a ligand of the B7 family member V-domain Ig suppressor of T-cell activation (VISTA, also known as PD-1H),^20^ which has been revealed to have inhibitory effects on human T cell function.^21^ IgSF11 has also been characterized as a gene frequently upregulated in intestinal type gastric cancers.^22^ Although IgSF11 has been revealed to function as a CAM, IgSF11 has a relatively long cytoplasmic tail (167 amino acids),^23^ raising the possibility that it possesses functions beyond cell adhesion. Indeed, IgSF11 contains a PDZ-binding motif at its C-terminus and has been demonstrated to interact with intracellular proteins through its PDZ-binding motif, promoting synaptic transmission and plasticity in neurons.^17^ This evidence suggests that the C-terminal cytoplasmic region of IgSF11 has a particular function.

In the present study, we identified a specific requirement for IgSF11 that has no previously reported function in the bone, as a regulator of osteoclast differentiation through interaction with PSD-95, a scaffold protein with multiple protein interaction domains. We report here that IgSF11 expression is induced by RANKL stimulation, and IgSF11 gene deletion results in increased bone mass due to impaired osteoclast differentiation but not by altering osteoblast activities. Using in vitro osteoclast culture systems, we demonstrate that IgSF11 homophilic interactions are required for proper osteoclast differentiation. Additionally, we show that impaired IgSF11-deficient osteoclast differentiation is restored by full-length IgSF11, which can associate with endogenous PSD-95, but is not restored by an IgSF11 mutant in which the 75 C-terminal amino acids required for association with PSD-95 have been truncated. RNAi-mediated gene knockdown of PSD-95 inhibited osteoclast differentiation, suggesting an important role for PSD-95 in osteoclast differentiation. These results demonstrate that IgSF11 controls osteoclast differentiation through association with intracellular molecules via its 75 C-terminal amino acids and suggest a role for IgSF11 not only as a cell adhesion molecule but also as a mediator of cell signaling during osteoclast differentiation.

## Results

### Identification of IgSF11 as an osteoclast differentiation-associated gene

**Fig. 1:**
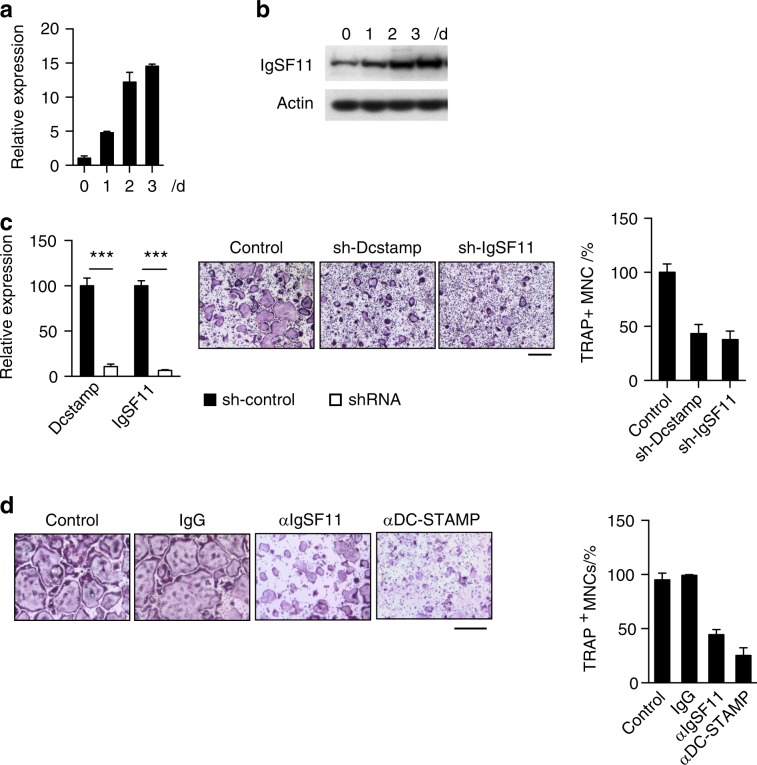
Identification of IgSF11 as an osteoclast differentiation-associated gene. **a** IgSF11 message expression during osteoclast differentiation. Total RNA was isolated from BMMs cultured with M-CSF + RANKL for the indicated days and used for Q-PCR. **b** IgSF11 protein expression during osteoclast differentiation. Total cell lysates were prepared from BMMs cultured with M-CSF + RANKL for the indicated days and used for western blotting with the indicated antibodies. **c** Effect of IgSF11 RNAi on osteoclast differentiation. BMMs retrovirally transduced with the indicated shRNAs were cultured with M-CSF + RANKL for three days. Relative expression of DC-STAMP and IgSF11 was determined by Q-PCR (left). Cells were stained for TRAP (middle). The frequency of TRAP^+^ multinucleated cells is shown (right). The scale bar represents 100 μm. **d** Effect of antibodies on osteoclast differentiation. BMMs were cultured with M-CSF + RANKL for three days in the presence of the indicated antibodies (left). The frequency of TRAP^+^ multinucleated cells (3 nuclei or more per cell) is shown (right). The scale bar represents 100 μm. Data are shown as the mean ± S.D. ***P < 0.001

We previously performed comparative gene expression profiling to identify novel gene targets associated with the maturation/late stage of osteoclast differentiation by using multinucleation as a functional proxy.^24^ As a result, we identified the gene *Igsf11*, which encodes the protein IgSF11, a member of the coxsackievirus and adenovirus receptor(CAR) subgroup of the CTX (the cortical thymocyte marker in Xenopus) family of transmembrane immunoglobulin-like CAMs (Ig-CAMs) (Fig. S1). IgSF11 shares an identical overall domain organization with a membrane-distal V-type domain and a membrane-proximal C2-type domain with coxsackievirus and adenovirus receptor (CAR), endothelial cell-selective adhesion molecule (ESAM), and CAR-like membrane protein (CLMP).^25–27^ To investigate the expression dynamics of IgSF11 during osteoclast differentiation, we generated osteoclasts in vitro from mouse bone marrow-derived monocytes (BMMs) treated with M-CSF + RANKL for up to three days and performed temporal Q-PCR-based expression analysis and western blotting. The expression of IgSF11 gradually increased during culture and peaked on day two after RANKL treatment at both message (Fig. 1a) and protein levels (Fig. 1b).

To investigate the role of IgSF11 in osteoclasts, we first performed RNAi experiments using retrovirus encoding shRNA specific to IgSF11. BMMs retrovirally transduced with shRNA against IgSF11 exhibited a reduction in tartrate-resistant acidic phosphatase-positive (TRAP^+^) multinucleated cells (i.e., mature osteoclasts) similar to BMMs transduced with shRNA against DC-STAMP, a known positive regulator of osteoclast maturation^28^ (Fig. 1c). We next examined the effect of targeting IgSF11 protein directly during osteoclast differentiation by using an anti-IgSF11 antibody. Control IgG and anti-DC-STAMP antibodies were used as negative and positive controls, respectively. The addition of either anti-IgSF11 or anti-DC-STAMP caused a significant reduction in TRAP^+^ multinucleated cells (Fig. 1d). These results suggest a potential regulatory role for IgSF11 in osteoclast maturation.

### IgSF11 regulates osteoclast differentiation and bone metabolism

To better understand the role of IgSF11 in osteoclasts and its function in bone remodeling, we generated IgSF11-deficient (IgSF11^−/−^) mice using clustered regularly interspersed short palindromic repeats (CRISPR)/Cas9 technology (Fig. S2a). We obtained gene knockout mice carrying a deletion of 48 base pairs (bp) (via a 55 bp deletion and 7 bp insertion event) in exon 1 of *Igsf11* (Fig. S2b). Mouse genotypes were verified by PCR (Fig. S2c), and the gene deletion was confirmed by western blotting (Fig. S2d). Similar to previous reports, male IgSF11^−/−^ mice were found to exhibit smaller testis and infertility,^19^ albeit with no apparent differences in body weight or size observed between IgSF11^+/+^ and IgSF11^−/−^ mice (Fig. S2e and data not shown). No generative dysfunction was observed in IgSF11^+/−^ males or IgSF11^−/−^ females^19^ (data not shown).

**Fig. 2:**
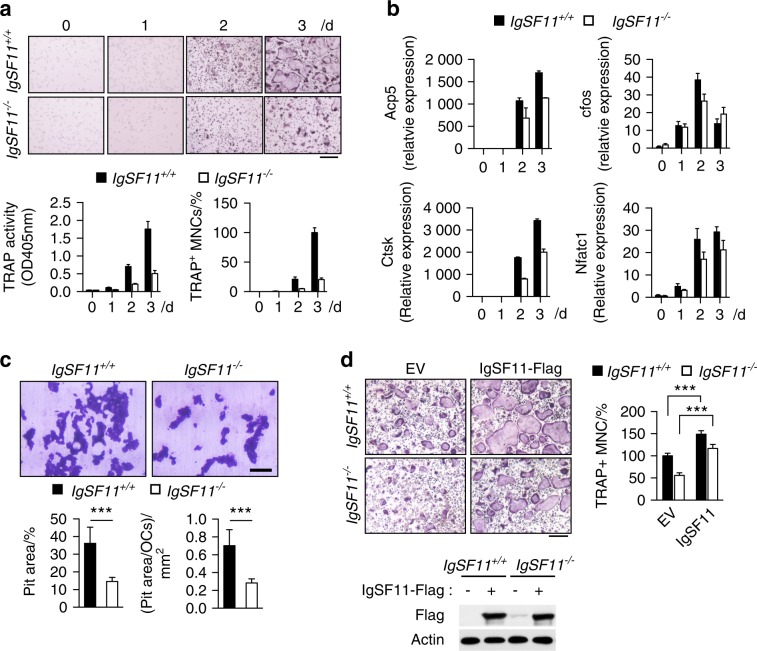
IgSF11 regulates in vitro osteoclast differentiation. **a** Osteoclast differentiation of IgSF11^+/+^ and IgSF11^−/−^ cells. BMMs were cultured with M-CSF + RANKL for the indicated days (top). TRAP activity and frequency of TRAP^+^ multinucleated cells (3 nuclei or more per cell) are shown (bottom). The scale bar represents 100 μm. **b** Gene expression during osteoclast differentiation. Total RNA was collected from IgSF11^+/+^ and IgSF11^−/−^ cultured cells, and the levels of the indicated genes were measured by Q-PCR. **c** Bone resorption activity of IgSF11^+/+^ and IgSF11^−/−^ osteoclasts. IgSF11^+/+^ and IgSF11^−/−^ BMMs were cultured with M-CSF + RANKL for three days, harvested, and recultured on dentin slices. The resorption area and pit are shown per cell. The scale bar represents 100 μm. **d** Osteoclast differentiation rescued by retroviral transduction of IgSF11 in IgSF11^−/−^ BMMs. BMMs were retrovirally transduced with empty vector (EV) or Flag-tagged IgSF11 expression vector followed by culture with M-CSF + RANKL for three days. The frequency of TRAP^+^ multinucleated cells (3 nuclei or more per cell) is shown. Expression of exogenous IgSF11 was confirmed by western blotting with an anti-Flag antibody. The scale bar represents 100 μm. Data are shown as the mean ± S.D. ***P < 0.001

To determine the specific cell-intrinsic role of IgSF11, we isolated BMMs from IgSF11^+/+^ and IgSF11^−/−^ mice and cultured them with M-CSF + RANKL to generate osteoclasts. We found significant reductions in total TRAP activity and in the frequency of multinucleated TRAP^+^ cells in IgSF11^−/−^ cultures compared to IgSF11^+/+^ cultures (Fig. 2a). No significant difference in osteoclast survival was observed (Fig. S3). Message levels of markers of differentiated osteoclasts, *Acp5* and *Ctsk*, were also reduced in IgSF11^−/−^ cultures (Fig. 2b). However, signaling pathways involved in early differentiation, such as expression/activation of important transcription factors (c-fos, NFATc1, and NF-κB) and activation of ITAM signaling (Syk, and PLCγ2), seemed to be comparable between IgSF11^+/+^ and IgSF11^−/−^ cells (Fig. 2b and Fig. S4). Similar to the reduction in osteoclast markers, we found significant reductions in overall resorption areas and in pit area per osteoclast in IgSF11^−/−^ cultures (Fig. 2c). Retroviral transduction of IgSF11^−/−^ BMMs with full-length IgSF11 completely rescued the impaired TRAP^+^ multinucleated cell formation, and furthermore, transduction of IgSF11^+/+^ BMMs with IgSF11 significantly enhanced TRAP^+^ multinucleated cell formation (Fig. 2d). These results suggest that IgSF11 positively regulates osteoclast differentiation and that the phenotypes observed in IgSF11^−/−^ osteoclast cultures can be attributed to deletion of the *Igsf11* gene.

**Fig. 3:**
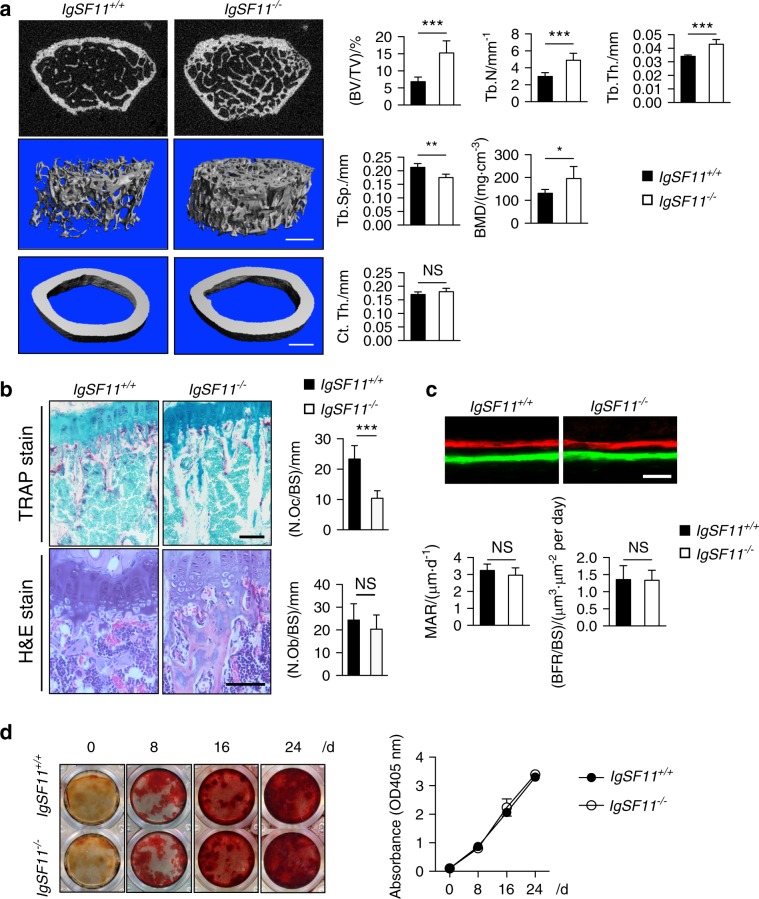
IgSF11 deficiency results in increased bone mass in mice. **a** Microcomputed tomography (μCT) images of femurs from IgSF11^+/+^ and IgSF11^−/−^ mice. The femurs of 12-week-old male mice were analyzed. Bone volume per tissue volume (BV/TV), trabecular thickness (Tb.Th), trabecular number (Tb.N), trabecular spacing (Tb.Sp), bone mineral density (BMD), and cortical thickness (Ct.Th) are shown. Scale bars represent 0.5 mm. **b** Histological analysis of tibias from 12-week-old IgSF11^+/+^ and IgSF11^−/−^ mice. Tibial sections were stained with TRAP or H&E. Osteoclast number per bone surface (N.Oc/BS) and osteoblast number per bone surface (N.Ob/BS) are shown. Scale bars represent 100 μm. **c** Dynamic histomorphometry of tibias from 12-week-old IgSF11^+/+^ and IgSF11^−/−^ mice. Mineral apposition rate (MAR) and bone formation (BFR) are shown. The scale bar represents 50 μm. **d** IgSF11^+/+^ and IgSF11^−/−^ bone marrow-derived stromal cells (BMSCs) were cultured with osteogenic medium for the indicated days and then stained with alizarin red. Data are shown as the mean ± S.D. *P < 0.05, **P < 0.01, ***P < 0.001

We next examined whether IgSF11 deficiency affects bone development and homeostasis. By immunohistochemistry of bone sections of mice in which osteoclasts and osteoblasts were labeled with red and cyan fluorescent proteins, respectively (TRAP-tdTomato/Col2.3-ECFP),^29^ we found IgSF11 protein expression in red^+^ (osteoclast) cells (Fig. S5a). In contrast, IgSF11 was barely detected in cyan^+^ (osteoblast) cells (Fig. S5a) or in bone matrix-embedded cells (osteocytes, data not shown). These results suggested that IgSF11 is expressed in osteoclasts among bone cells in vivo. We confirmed no detection of IgSF11 in bone sections of IgSF11^−/−^ mice (Fig. S5b). We then prepared gender- and age-matched IgSF11^+/+^ and IgSF11^−/−^ mice and performed representative 3D reconstructions of trabecular bone. Bone microstructure imaging by high-resolution microcomputed tomography (μCT) of IgSF11^−/−^ male mice revealed significantly increased bone mass, characterized by increased bone indices that included trabecular bone volume per tissue volume (BV/TV), trabecular number (Tb.N), trabecular thickness (Tb.Th), and bone mineral density (BMD), with concomitant decreases in trabecular spacing (Tb.Sp) (Fig. 3a). No significant differences in cortical bone thickness (Ct.Th) were detected between IgSF11^+/+^ and IgSF11^−/−^ mice (Fig. 3a). A similar tendency toward increased bone mass was also observed in IgSF11^−/−^ female mice (Fig. S6). TRAP-stained IgSF11^−/−^ bone sections revealed a significant reduction in the number of osteoclasts on the bone surface (N.Oc/BS) compared to the number in IgSF11^+/+^ sections (Fig. 3b). In contrast, quantitation of the osteoblast number per bone surface (N.Ob/BS) revealed normal numbers of osteoblasts in bone sections from IgSF11^−/−^ mice (Fig. 3b). Serum levels of the bone resorption markers C-terminal telopeptide of type I collagen (CTX-I) and tartrate-resistant acid phosphatase 5b (TRACP-5b) were both found to be reduced in IgSF11^−/−^ mice (Fig. S7). In contrast, serum levels of osteocalcin, a bone formation marker, showed no significant differences between IgSF11^+/+^ and IgSF11^−/−^ mice (Fig. S7). Dynamic histomorphometry by sequential injections of calcein and xylenol orange revealed normal bone formation rates in IgSF11^−/−^ mice (Fig. 3c). In addition, in vitro osteogenic differentiation exhibited no differences in IgSF11^−/−^ calvaria-derived osteoblast precursors compared to those from IgSF11^+/+^ calvaria as visualized by alizarin red staining for mineralized nodule formation and calcium deposits (Fig. 3d). These results suggest that IgSF11 deficiency results in increased bone mass due to impaired osteoclast differentiation but not impaired osteoblast development or function.

### IgSF11 functions through homophilic interactions

**Fig. 4:**
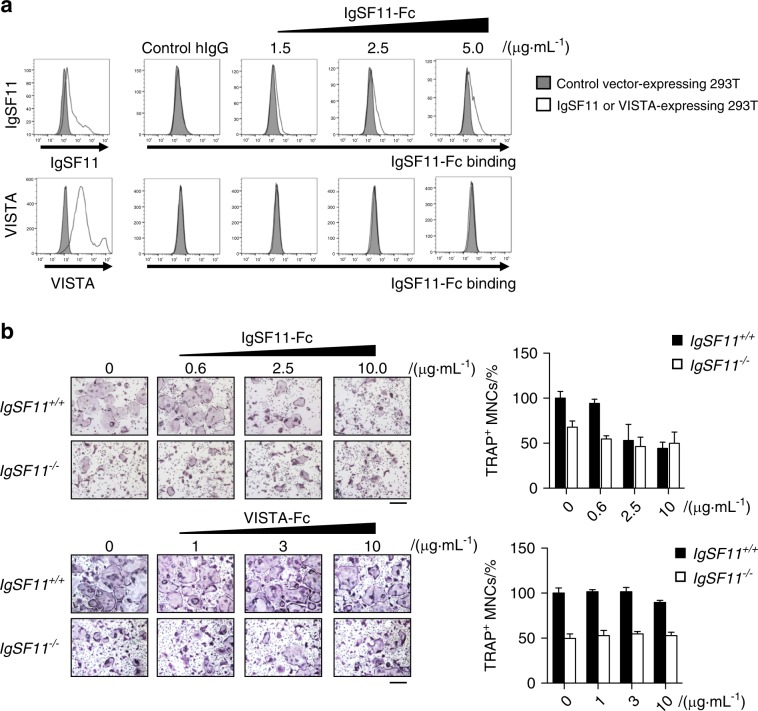
IgSF11 functions through homophilic interactions. **a** Staining of IgSF11- or VISTA-expressing 293 T cells with IgSF11-Fc protein. 293 T cells were transiently transfected with N-terminal Flag-tagged IgSF11 or VISTA expression vector and incubated with the indicated concentration of IgSF11-Fc, followed by staining with anti-human IgG-PE antibody. Control human IgG was used as a negative control. Surface expression of IgSF11 and VISTA was confirmed by staining with anti-Flag or anti-VISTA antibodies, respectively. **b** Effects of recombinant IgSF11 or VISTA proteins on osteoclast differentiation. IgSF11^+/+^ and IgSF11^−/−^ BMMs were cultured with M-CSF + RANKL plus the indicated concentration of recombinant protein for three days. The frequency of TRAP^+^ multinucleated cells (3 nuclei or more per cell) is shown. Scale bars represent 100 μm

Having shown that IgSF11 regulates bone metabolism through regulation of osteoclast differentiation, we sought to identify the molecular mechanism that regulates IgSF11-mediated osteoclast differentiation. IgSF11 has been reported to mediate cell–cell adhesion as a CAM.^15^ We found that RANKL-treated BMMs expressing EGFP-tagged IgSF11 showed accumulation of EGFP at cell–cell contacts (Fig. S8a), suggesting the involvement of IgSF11 in intercellular interactions during osteoclast differentiation. IgSF11 has been reported to engage in both homophilic interactions^15^ and in heterophilic interactions with VISTA.^20,21^ By flow cytometric analysis, we found VISTA expression on both IgSF11^+/+^ and IgSF11^−/−^ BMMs (Fig. S8b), raising the potential for involvement of VISTA in osteoclast differentiation through an interaction with IgSF11. To determine the type of of interaction involved in osteoclast differentiation, we generated a recombinant soluble IgSF11-Fc protein that consisted of the extracellular region of IgSF11 fused to the human IgG Fc region. We performed binding assays using IgSF11-Fc, which revealed dose-dependent binding of IgSF11-Fc to IgSF11-expressing 293 T cells but not to VISTA-expressing 293 T cells (Fig. 4a). We also found that treatment with IgSF11-Fc significantly reduced RANKL-induced formation of TRAP^+^ multinucleated cells in IgSF11^+/+^ cultures in a dose-dependent manner, whereas no inhibitory effect of IgSF11-Fc was observed in IgSF11^−/−^ cultures (Fig. 4b). In contrast, when we examined the effect of recombinant soluble VISTA-Fc protein on the formation of TRAP^+^ multinucleated cells, no apparent effect was observed in IgSF11^+/+^ or IgSF11^−/−^ cultures (Fig. 4b). These results show that IgSF11-Fc perturbs osteoclast differentiation and suggest that IgSF11-mediated osteoclast differentiation depends on an IgSF11-IgSF11 homophilic interaction.

### The PSD-95-associated region of IgSF11 is required for osteoclast differentiation

**Fig. 5:**
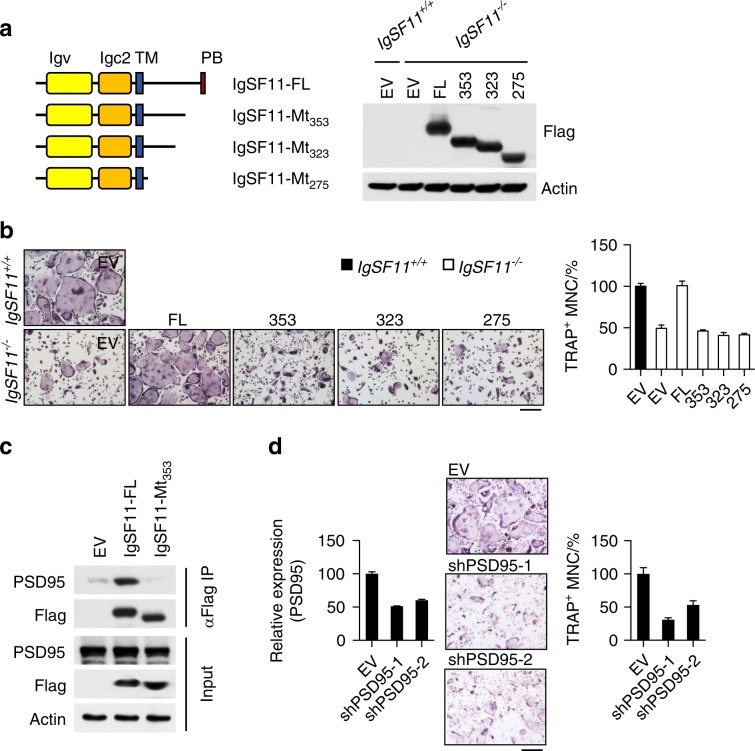
IgSF11-PSD-95 protein complex formation is required for IgSF11-mediated regulation of osteoclast differentiation. **a** Schema of IgSF11 wild type and IgSF11 deletion mutants to be used for retroviral transduction of IgSF11^−/−^ BMMs. All constructs were Flag-tagged at the C-terminus (left). IgSF11^+/+^ BMMs were used as controls. Protein expression was confirmed by western blotting with an anti-Flag antibody (right). PB, PDZ-binding domain. **b** The cells prepared in **a** were cultured with M-CSF + RANKL and stained for TRAP. The frequency of TRAP^+^ multinucleated cells (3 nuclei or more per cell) is shown. Scale bars represent 100 μm. **c** Coimmunoprecipitation of IgSF11 with PSD-95. IgSF11^−/−^ BMMs retrovirally transduced with the indicated vectors were lysed and immunoprecipitated with anti-Flag antibody, and western blotting was performed with the indicated antibodies. **d** Effect of PSD-95 RNAi on osteoclast differentiation. BMMs retrovirally transduced with the indicated shRNAs were cultured with M-CSF + RANKL for three days. The relative expression of PSD-95 was determined by Q-PCR. Cells were stained for TRAP. The frequency of TRAP^+^ multinucleated cells is shown. The scale bar represents 100 μm. Data are shown as the mean ± S.D.

We sought to further investigate the mechanism by which IgSF11 regulates osteoclast differentiation. To gain insight into IgSF11 function, we generated mutant expression vectors lacking various sections of the IgSF11 intracellular region (IgSF11-Mt_275_ lacks the entire intracellular region, IgSF11-Mt_323_ lacks 105 amino acids from the C-terminus, and IgSF11-Mt_353_ lacks 75 amino acids from the C-terminus) (Fig. 5a). IgSF11^−/−^ BMMs were retrovirally transduced with full-length IgSF11 (IgSF11-FL) or mutant vectors and cultured with M-CSF + RANKL to generate osteoclasts. Compared to the full recovery of TRAP^+^ multinucleated cell numbers by IgSF11-FL, IgSF11-Mt_275_ failed to rescue the formation of TRAP^+^ multinucleated cells (Fig. 5b), suggesting a critical role for the IgSF11 intracellular region during osteoclast differentiation. Both IgSF11-Mt_323_ and IgSF11-Mt_353_ mutants also failed to restore TRAP^+^ multinucleated cells (Fig. 5b), suggesting a specific requirement for the 75 C-terminal amino acids of IgSF11 for osteoclast differentiation.

It has been reported that IgSF11 interacts with PSD-95^17^ through the IgSF11 C-terminal PDZ-binding motif (PB). PSD-95 is a specialized scaffold protein with multiple protein interaction domains and forms the backbone of an extensive postsynaptic protein complex that organizes receptors and signal transduction molecules at the synaptic contact zone.^30^ To investigate the involvement of PSD-95 in osteoclast differentiation, we tested whether IgSF11 forms a signaling complex with PSD-95 in osteoclasts. For this, we retrovirally transduced flag-tagged full-length IgSF11 or the C-terminal deletion mutant form of IgSF11 into IgSF11-deficient cells and performed coimmunoprecipitation to examine whether IgSF11 associates with PSD-95. We found, as predicted, that IgSF11-FL associates with PSD-95 (Fig. 5c). In contrast, an association of IgSF11-Mt_353_ with PSD-95 was not observed. Furthermore, we performed RNAi-mediated gene knockdown of PSD-95 in osteoclasts. Wild-type BMMs retrovirally transduced with shRNA against PSD-95 exhibited significant reductions in the frequency of multinucleated TRAP^+^ cells (Fig. 5d). These results suggest that PSD-95 plays a role in osteoclasts and that the association of IgSF11 with PSD-95 through the 75 amino acids at the IgSF11 C-terminus is specifically required for proper osteoclast differentiation.

## Discussion

During osteoclast differentiation, RANKL stimulation drives the commitment of osteoclast precursors to become large multinucleated mature osteoclasts. In addition to RANKL stimulation, costimulation and cell–cell interactions through surface receptors are further required to provide additional signaling necessary for osteoclast maturation. In this study, we revealed that IgSF11, a member of the Ig-CAM family, engages in homophilic interactions and leads to increased osteoclast differentiation through association with PSD-95. Our findings reveal a critical role for IgSF11 in osteoclast differentiation.

We showed that IgSF11 is involved in osteoclast differentiation and maturation. Although we cannot rule out a role for IgSF11 during the early stage of differentiation characterized by TRAP^+^ mononuclear preosteoclasts, most of the early stage signaling pathways, such as NFATc1, c-fos and NF-κB, seemed to be unaffected. In contrast, the difference between IgSF11^+/+^ and IgSF11^−/−^ cultures was more obvious when we counted TRAP^+^ large multinucleated osteoclasts. Hence, IgSF11 deficiency is likely to have a more significant effect on the process of osteoclast maturation required for the formation of multinucleated giant cells.

We sought to identify the molecular mechanisms by which IgSF11 regulates osteoclast differentiation by generating mutant expression vectors lacking various sections of the IgSF11 intracellular region and identified that the region encompassing the 75 C-terminal amino acids of IgSF11 is important for osteoclast differentiation. We also revealed that PSD-95 is involved in osteoclast differentiation and that IgSF11 associates with PSD-95 through the C-terminal 75 amino acid region and forms a protein complex in osteoclasts. Given that PSD-95 forms the backbone of a protein complex in the nervous system,^31^ it is plausible that the IgSF11-PSD-95 protein complex plays a role in the regulation of signal transduction at the cell–cell contact site during osteoclast differentiation. Indeed, PSD-95 is known to interact with a large number of proteins, including Src family kinases, through its three PDZ domains, SH3 domain, and/or guanylate kinase domain and can also multimerize to form an extended scaffold.^32,33^ It is also possible that the IgSF11 C-terminal region recruits additional proteins and/or signaling molecules. Further study is needed to clarify the mechanism underlying IgSF11-mediated regulation of osteoclast differentiation.

IgSF11 has been shown to engage in homophilic interactions as well as in heterophilic interactions, such as with VISTA,^21^ which is involved in T cell activation.^34,35^ Although we observed expression of VISTA on BMMs and preosteoclasts (Fig. S8b), we cannot conclude that IgSF11-VISTA interactions are operative in osteoclast differentiation given that (1) we found neither inhibitory nor stimulatory effects of functional VISTA-Fc on osteoclast differentiation, (2) IgSF11-Fc inhibited IgSF11^+/+^ osteoclast differentiation to the same degree as that of IgSF11^−/−^ osteoclasts, and (3) IgSF11-Fc showed no inhibitory effects on IgSF11^−/−^ culture. We did not find heterophilic interactions between IgSF11 and VISTA. The discrepancy between previous studies and this study may be explained by differences in experimental systems, different recombinant IgSF11 proteins, and a different binding assay system. Nevertheless, the recombinant IgSF11-Fc used in this study was functionally active with respect to its effects on osteoclast differentiation (Fig. 4b). These results suggest that IgSF11 regulates osteoclast differentiation through homophilic interactions.

Here, we demonstrated that intracellular region-truncated mutants (IgSF11-Mt_257_, IgSF11-Mt_323_, and IgSF11-Mt_353_; in all the extracellular region was intact) failed to rescue the impaired osteoclast differentiation in IgSF11^−/−^ cultures. These results imply that although the cell–cell adhesion function mediated by IgSF11 through homotypic interactions may be important in some contexts, it does not appear to be essential for osteoclast differentiation. Rather, signaling mediated through the IgSF11 intracellular region by IgSF11 homotypic interactions is more likely required for osteoclast differentiation. IgSF11 might function not just as a CAM but as a mediator of signaling necessary for osteoclast differentiation.

Bone remodeling is carried out by tightly controlled coupling of osteoclastic bone destruction and osteoblastic bone formation.^1,36^ This coupling is key to preserving bone architecture and strength. Current treatments for bone loss, such as bisphosphonates and anti-RANKL antibody (Denosumab), primarily target early osteoclast commitment and/or osteoclast viability.^37^ However, these treatments often fail to uncouple bone degradation and formation and result in compromised bone strength due to unintended inhibition of coupled bone formation.^38,39^ We focused on IgSF11 in a study aiming to identify gene targets associated with the maturation/late stage of osteoclast, which we believe will identify better treatment targets (inhibit osteoclast resorption without preventing contribution to production bone formation),^24^ and we showed that IgSF11 deficiency uncoupled bone degradation and formation and impaired osteoclast-mediated bone resorption without affecting osteoblast-mediated bone formation. These results suggest that IgSF11 might be a promising target for selectively inhibiting bone loss.

In conclusion, we identified a previously unknown function of IgSF11 as a regulator of osteoclast differentiation. Identification and characterization of additional key regulators of osteoclast differentiation would aid in the development of therapeutic strategies for the treatment of skeletal diseases. Taken together, these results demonstrate IgSF11 as a regulator of osteoclast differentiation.

## Materials and methods

### Mice

IgSF11^−/−^ mice were generated using CRISPR/Cas9 technology with the cooperation of NPO Biotechnology Research and Development.^40^ A small guide RNA (sgRNA) targeting IgSF11 exon 1 was synthesized in vitro. C57BL/6 female mice were superovulated, and oocytes were collected from the ampullae and then coincubated with sperm from C57BL/6 male mice. Pronuclear stage eggs were injected with pX330 plasmids containing hCas9 and sgRNA (5′- GGCGGCGCTCCGCTCCGGCGTCCTGG-3′) at 5 ng·mL^−1^ using a micromanipulator. The fertilized eggs were transferred into the oviducts of pseudopregnant ICR females, and F0 generation mice were born. F0 offspring were genotyped using PCR amplification and subsequent direct sequencing. Five of the seven offspring carried a nonmosaic genomic mutation. We bred one of these mice carrying a heterozygous 48 bp deletion mutation with the C57BL/6 background strain. Homozygous mutant mice were bred from F1 heterozygotes. All neonatal mice were genotyped by sanger sequencing using the following primers: 5′- GTTCTGTGCATTCTGCGGCT-3′ (sense) and 5′-TATCGCAAACTCCTTCAAGGCT-3′ (antisense), which generated products of 625 bp for the wild-type allele and 577 bp for the mutant allele, and/or polymerase chain reaction (PCR) using the following primers: 5′-GCTTGGTCCCACGCTGCACC-3′ (sense) and 5′-CTCACCGAGCAGCGACACGA-3′ (antisense), which generated product of 125 bp for the wild-type allele and 77 bp for the mutant allele. In each experiment, homozygous IgSF11^+/+^ and IgSF11^−/−^ littermate mice that were generated by intercrossing heterozygous mice were compared. All mice were maintained and used in accordance with guidelines approved by the Institutional Animal Care and Use Committee (IACUC) at the University of Pennsylvania.

### Reverse transcription and real-time PCR (Q-PCR)

Total RNA was extracted from cells using TRIzol reagent (Invitrogen), and 1μg–5 μg of total RNA was reverse transcribed using random hexamer primers and SuperScript III reverse transcriptase (Invitrogen). cDNA corresponding to 10 ng of total RNA was analyzed by Q-PCR using a QuantStudio3 (Applied Biosystems) and the following specific TaqMan® probes: IgSF11 (Mm00464360_m1), ACP5 (Mm00475698_m1), DC-STAMP (Mm01168058_m1), Ctsk (Mm00484036_m1), PSD-95 (Mm00492193_m1), and 18 S (Hs99999901_s1). The CT method of relative quantification was used to determine the fold change in expression.

### Western blotting

BMMs and osteoclast cultures were washed with ice-cold phosphate-buffered saline (PBS) and lysed with ice-cold radio immunoprecipitation (RIPA) lysis buffer (20 mmol·L^−1^ Tris-HCl, pH 7.5, 150 mmol·L^−1^ NaCl, 1% NP-40, 0.5% sodium deoxycholate, 1 mmol·L^−1^ EDTA, 0.1% SDS, protease and phosphatase inhibitor cocktail (Roche)). The lysates were centrifuged to remove debris, and protein concentrations were determined using the Bradford assay. Equal amounts of lysates (2 μg–50 μg of protein) were fractionated by SDS–polyacrylamide gel electrophoresis (SDS–PAGE) on 4%–12% gradient gels and transferred onto polyvinyl difluoride (PVDF) membranes. Western blotting was performed with the following antibodies: anti-IgSF11 (2067 and 2068), which was a kind gift from Dr. Eunjoon Kim (KAIST, Korea), anti-p-IkBα: 2859, anti-Syk: 2712, anti-p-Syk: 2701, anti-PLCγ2: 3872, and anti-p-PLCγ2: 3874 (Cell Signaling Technology), anti-Flag: M2 (Sigma-Aldrich), anti-actin: sc-47778 (Santa Cruz Biotechnology), and anti-PSD-95: K28/43 (BioLegend).

### Microcomputed tomography

Femurs from 12-week-old IgSF11^+/+^ and IgSF11^−/−^ male mice and 16-week-old female mice were harvested, fixed for 1 h in 4% formalin, and then incubated with PBS. Scanning was performed using μCT (μCT35, SCANCO Medical AG, Brüttisellen, Switzerland). For trabecular bone analysis, scans were performed at the distal femoral metaphysis 0.4 mm proximal to the growth plate. For cortical bone, mid-diaphysis of femurs was scanned. All scans were performed at a resolution of 6 μm per slice using an X-ray energy of 55 kvp and an integration time of 300 ms. A total of 200 slices for trabecular bone and 50 slices for cortical bone were analyzed using the instrument’s software.

### Bone histology and histomorphometry

For histology and histomorphometry, 12-week-old IgSF11^+/+^ and IgSF11^−/−^ mice were perfused with 4% paraformaldehyde plus sucrose for fixation, and bone tissues were further fixed with 4% paraformaldehyde plus sucrose for 3 h at 4 °C. Bones were incubated with 10% EDTA for 2 weeks for decalcification and embedded in O.C.T. compound (Tissue-Tek). Longitudinal sections, 10 μm thick, were prepared using Kawamoto’s film method^41^ and stained with H&E for OB parameter analysis or stained with TRAP for OC parameter analysis within the primary and secondary spongiosa. Measurement of mineral apposition rate was performed as described previously.^42^ In brief, 12-week-old IgSF11^+/+^ and IgSF11^−/−^ mice were injected with calcein, then with xylenol orange 10 days later, and then killed 2 days after that. For immunohistochemistry, bone samples of 9-week-old IgSF11^+/+^/TRAP-tdTomato/Col2.3-ECFP mice, which were prepared by perfusion with 4% paraformaldehyde plus sucrose and further fixed with 4% paraformaldehyde plus sucrose for 16 h at 4 °C, were obtained and embedded in SCEM (SECTION-LAB). Longitudinal sections, 10 μm thick, were prepared using Kawamoto’s film method. Fluorescence and phase-contrast images were acquired using a laser-scanning confocal microscope (TCS SP8, Leica) and analyzed using NIS Elements software (NIKON). The following antibodies were used for blocking: goat F(ab) anti-mouse IgG H&L: ab6668 (Abcam) and anti-CD16/CD32 monoclonal antibody (93): 14-0161 (Thermo Fisher Scientific). The following antibodies and dye were used for staining: anti-IgSF11: sc-393816 (Santa Cruz Biotechnology), Alexa Fluor 488 goat anti-mouse IgG (H + L): A11029 (Thermo Fisher Scientific), and TO-PRO-3 iodide: T3605 (Thermo Fisher Scientific).

### In vitro osteoclast (OC) differentiation and tartrate-resistant acid phosphatase (TRAP) staining

BMMs and osteoclasts were prepared, as described previously.^43^ In brief, whole BM was extracted from the femurs and tibias of mice and incubated in 100 mm petri dishes in α-MEM containing 10% fetal bovine serum and M-CSF (5 ng·mL^−1^) overnight. Non-adherent cells were collected and cultured for 3 days with M-CSF (30 ng·mL^−1^) to generate BMMs. For OC differentiation, BMMs were plated at 5 × 10^3^ per well in 96-well cell culture plates and cultured with M-CSF (60 ng·mL^−1^) and RANKL (150 ng·mL^−1^) for 3 days. Osteoclasts were stained using the Acid, Phosphatase, Leukocyte (tartrate-resistant acid phosphatase) Kit (387A-1KT, Sigma) following the manufacturer’s instructions. To analyze bone pit formation, BMMs were cultured with M-CSF and RANKL for 3 days to differentiate into osteoclasts. Differentiated osteoclasts were harvested and seeded on dentin slices at 2 × 10^4^ per well in 96-well cell culture plates and cultured for 3 additional days. Cells on dentin slices were removed by washing with PBS and stained with 1% hematoxylin. Resorption pits were visualized under a light microscope. Pit area was measured by ImageJ (National Institutes of Health). Anti-DC-STAMP (MABF39) and anti-IgSF11 (sc-393816) antibodies were purchased from EMD Millipore, Santa Cruz Biotechnology, and BioLegend, respectively. Recombinant human IgSF11-Fc chimera protein was produced by Accurus Biosciences. To do so, cDNA from the reported clone GenBank AY358141 was modified to improve protein expression stability by deleting the two C-terminal amino acids and then subcloned in-frame with an N-terminal proprietary (Accurus) signal peptide, a C-terminal ECD linker, and the sequence for the human IgG1 Fc region. This construct was then stably transfected into CHO cells, and the secreted protein was purified. Recombinant mouse VISTA-Fc chimera protein (7005-B7) was purchased from R&D.

### In vitro osteogenic differentiation

The osteoblast differentiation assay was performed, as previously described.^44^ In brief, bone marrow stromal cells were seeded at 1 × 10^5^ cells/well in 48-well plates with osteogenic medium containing 10% FBS, 10 mmol·L^−1^ β-glycerophosphate, and ascorbic acid (100 μg·mL^−1^) for 24 days. Mineralized nodule formation was determined on days 8, 16, and 24 by staining with alizarin red S solution (ARS). ARS stock solution was prepared by dissolving 1 mg of alizarin red S in 100 mL of 1% KOH. At the designated time point (depending on the experiment) of a given culture in osteogenic induction medium, cells were washed, fixed, and then stained with 40 mmol·L^−1^ fresh ARS solution (pH 4.2). Calcium deposits were then visualized by red color under light microscopy. To quantify the staining, the ARS Staining Quantification Assay (ARed-Q) method (ScienCell Research Laboratories, Carlsbad, CA, USA) was used based on the manufacturer’s instructions. The desired ARS dye was quantified by measuring the absorbance at 405 nm.

### Flow cytometric analysis

In total 293 T cells transfected with expression vectors encoding IgSF11 (Flag-tagged at the N-terminus) or VISTA (Flag-tagged at the C-terminus) were washed with PBS and detached using enzyme-free cell dissociation buffer (Millipore). After preparing single-cell suspensions, cells were incubated with the indicated concentration of IgSF11-Fc or control human IgG for 30 min on ice. After washing, the cells were stained with PE-labeled goat anti-human IgG for 15 min on ice. After washing again, cells were stained with TO-PRO-3 (Life Technology, Inc.) to exclude dead cells and analyzed using an LSR (BD Bioscience) analyzer. Surface expression of IgSF11 and VISTA was confirmed using anti-Flag and anti-VISTA antibodies, respectively.

### Retrovirus preparation and transduction

Mouse IgSF11 cDNA was amplified by PCR with the following primers: a sense primer with the EcoRI site (5′-GGGAATTCAGTGGCCTCGGCGCTCCCGTGTCC-3′) and antisense primers with the XhoI site (5′-CTCGAGTACCAGGGACCCTGCTCGACTCTG-3′ for IgSF11-FL, 5′-AACTCGAGTGATGGGATAACTGCATT-3′ for IgSF11-Mt_353_, 5′-CTCGAGGTTGTTCCA GTATCGACTGTTGTA-3′ for IgSF11-Mt_323_, and 5′-CTCGAGCTCCTCCTCTTTGTTTTTGCTTCT-3′ for IgSF11-Mt_275_) and cloned into the EcoRI-XhoI fragment of the pMX-Flag vector to generate pMX vectors encoding C-terminally FLAG-tagged IgSF11-FL, IgSF11-Mt_353_, IgSF11-Mt_323_, and IgSF11-Mt_275._ To prepare retroviral particles, Plat-E packaging cells were plated on 100 mm culture dishes and cotransfected with pSuper vectors encoding siRNAs targeting IgSF11 (5′-AGTAATAGCCGGAGCGGTT-3′), DC-STAMP (5′-GAATGACACTAGAGGAGAA-3′), and PSD-95 (sh-1: 5′-GGAACAGCTTATGAATAGT-3′, and sh-2: 5′-GTTCACGGAGTGCTTCTCA-3′), and pMX vectors encoding C-terminally FLAG-tagged IgSF11-FL, IgSF11-Mt_353_, IgSF11-Mt_323_, and IgSF11-Mt_275_ using PEImax (Polysciences). Empty pSuper vector and pMX vector were used as negative controls. After 3 days, medium containing each retrovirus was harvested and passed through a syringe filter (0.45 μm pore diameter). BMMs were transduced with retroviruses for 16 h with hexadimethrine bromide (8 μg·mL^−1^) in the presence of M-CSF (60 ng·mL^−1^). After washing with fresh medium, infected cells were selected by culturing for 2 days in the presence of puromycin (2 μg·mL^−1^) and M-CSF (60 ng·mL^−1^). Puromycin-resistant BMMs were used for the experiments.

### Statistical analysis

All experiments were analyzed using one-way ANOVA or 2-tailed paired Student’s *t* test with Prism 7.0 (GraphPad Software). *P* < 0.05 was considered statistically significant.

## Supplementary information

Supplementary figure 8. IgSF11 localizes to cell–cell contacts

Supplemental information legends

Supplementary figure 1. Cloning of IgSF11 as a novel gene involved in late-stage osteoclast differentiation

Supplementary figure 2. Generation of IgSF11-deficient mice by CRISPR/Cas9 system

Supplementary figure 3. IgSF11 deficiency does not affect osteoclast survival

Supplementary figure 4. NF-κB and ITAM-signaling in IgSF11-deficient cells

Supplementary figure 5. Expression of IgSF11 in bone cells in vivo

Supplementary figure 6. Microcomputed tomography analysis of IgSF11<sup>-/-</sup> female mice

Supplementary figure 7. Serum levels of bone resorption and formation markers
