# Supplementary figures and images for: IgSF11 regulates osteoclast differentiation through association with the scaffold protein PSD-95
Source: Bone Res. 2020 Feb 10;8:5. doi: 10.1038/s41413-019-0080-9 (PMC7010662; doi:10.1038/s41413-019-0080-9)

Supplementary figure 8

**a**

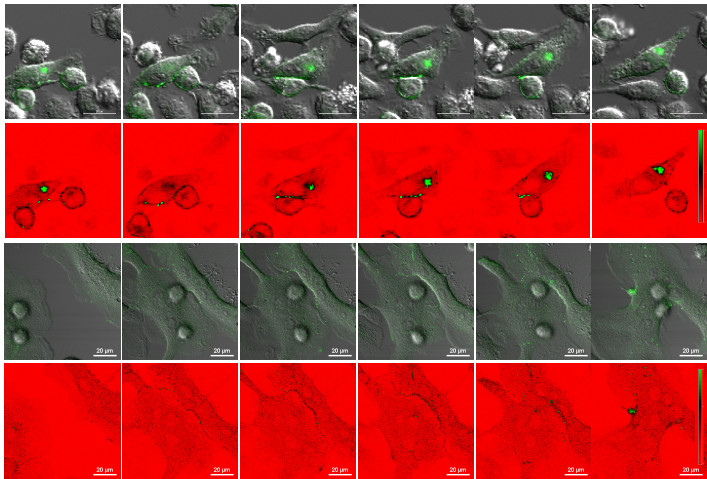

**b**

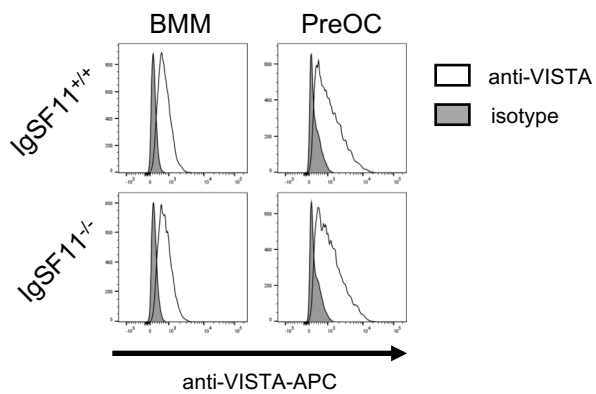

Supplement: Supplementary file 1 — Supplementary figure 8. IgSF11 localizes to cell–cell contacts [file 41413_2019_80_MOESM1_ESM.pdf]

Supplementary figure 2

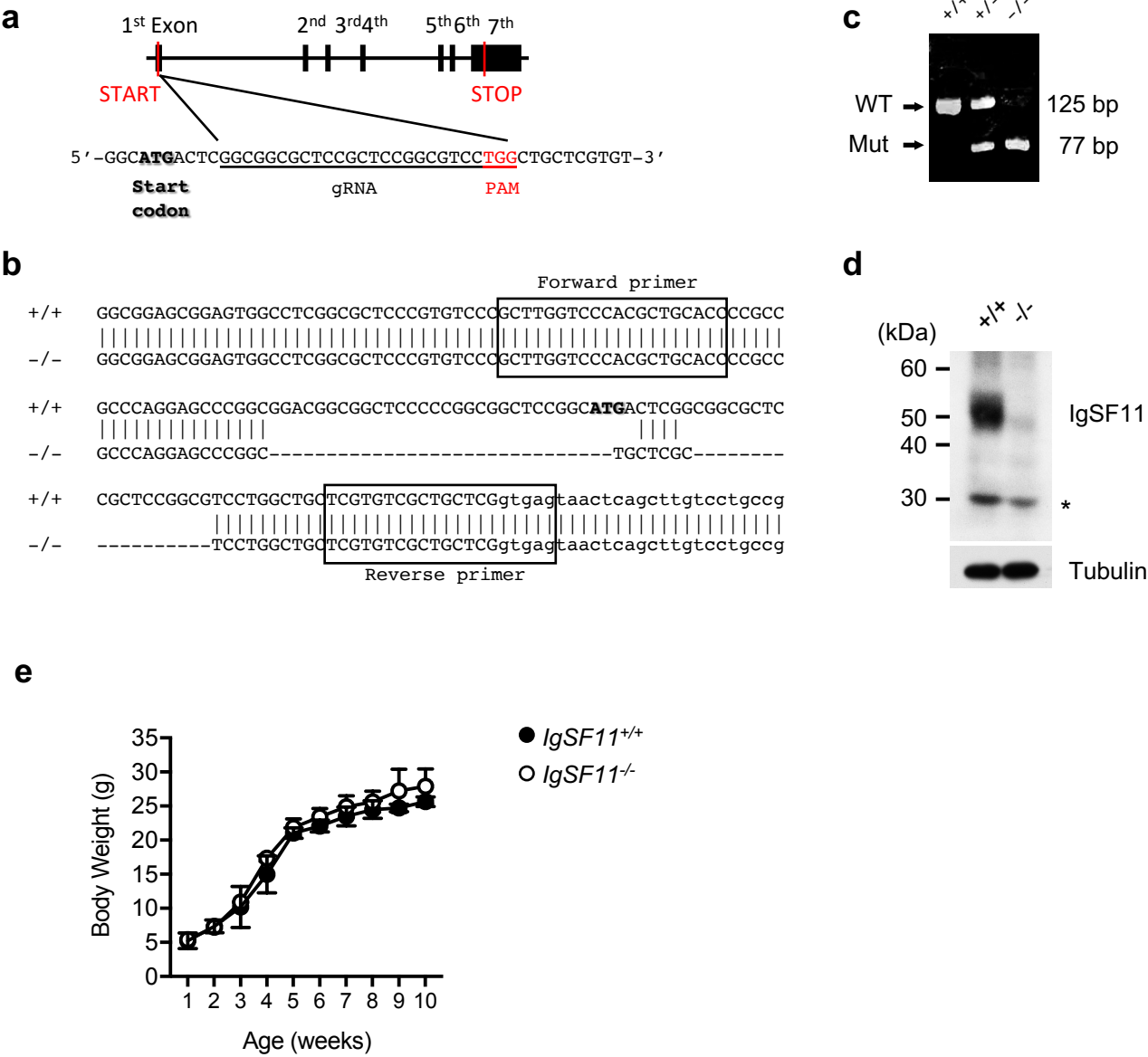

Supplement: Supplementary file 4 — Supplementary figure 2. Generation of IgSF11-deficient mice by CRISPR/Cas9 system [file 41413_2019_80_MOESM4_ESM.pdf]

Supplementary figure 3

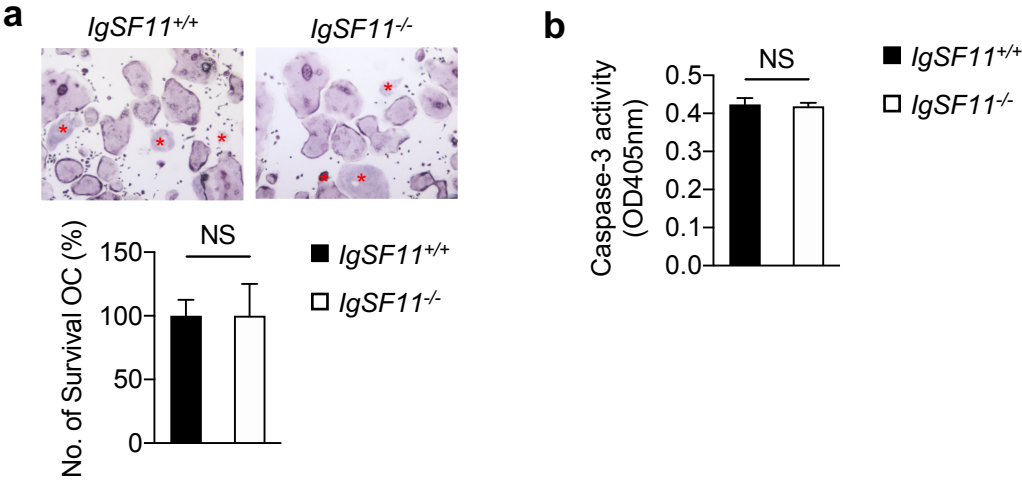

Supplement: Supplementary file 5 — Supplementary figure 3. IgSF11 deficiency does not affect osteoclast survival [file 41413_2019_80_MOESM5_ESM.pdf]

Supplementary figure 4

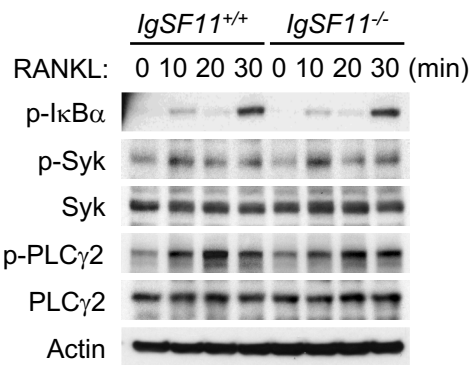

Supplement: Supplementary file 6 — Supplementary figure 4. NF-κB and ITAM-signaling in IgSF11-deficient cells [file 41413_2019_80_MOESM6_ESM.pdf]

**a**

*IgSF11*<sup>+/+</sup>/TRAP-tdTomato/Col2.3-ECFP

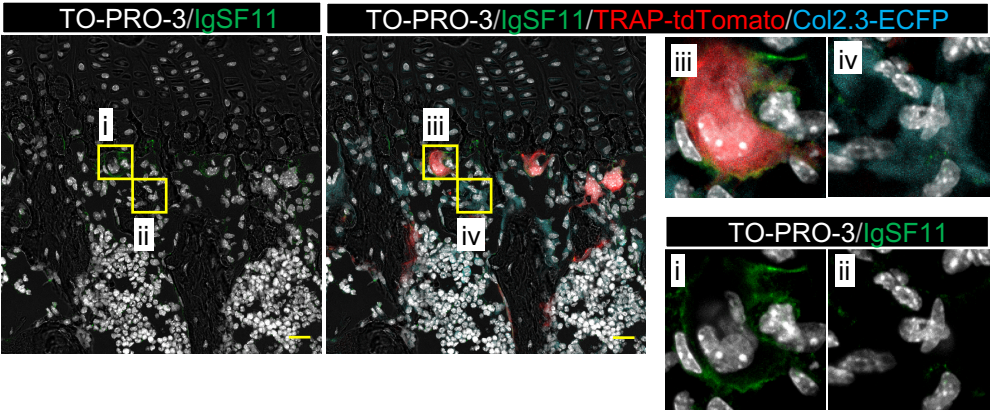

**b**

*IgSF11*<sup>+/+</sup>

*IgSF11*<sup>-/-</sup>

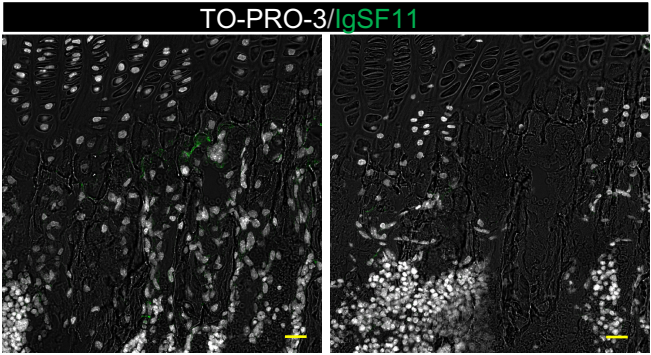

Supplement: Supplementary file 7 — Supplementary figure 5. Expression of IgSF11 in bone cells in vivo [file 41413_2019_80_MOESM7_ESM.pdf]

Supplementary figure 6

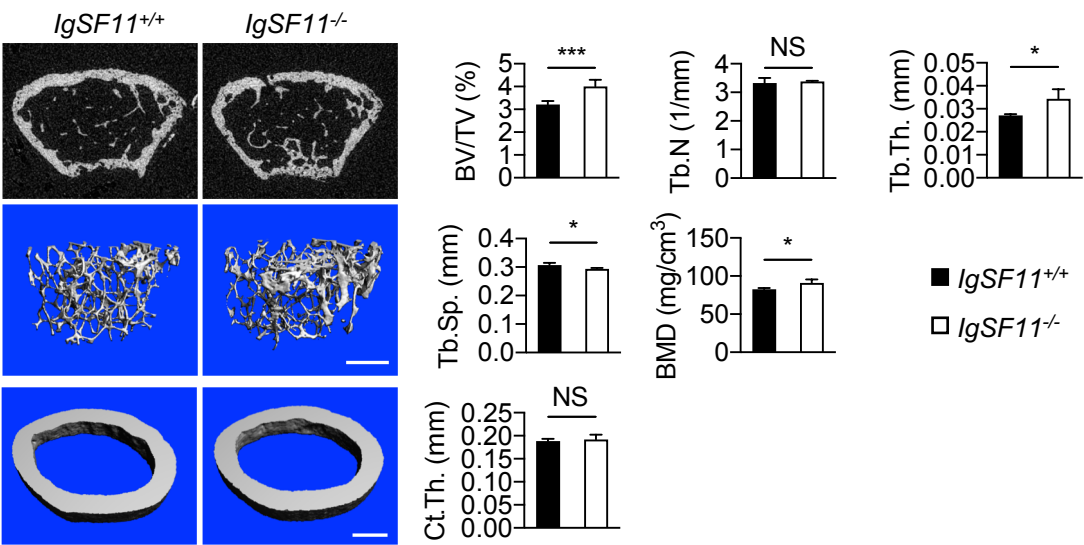

Supplement: Supplementary file 8 — Supplementary figure 6. Microcomputed tomography analysis of IgSF11-/- female mice [file 41413_2019_80_MOESM8_ESM.pdf]

Supplementary figure 7

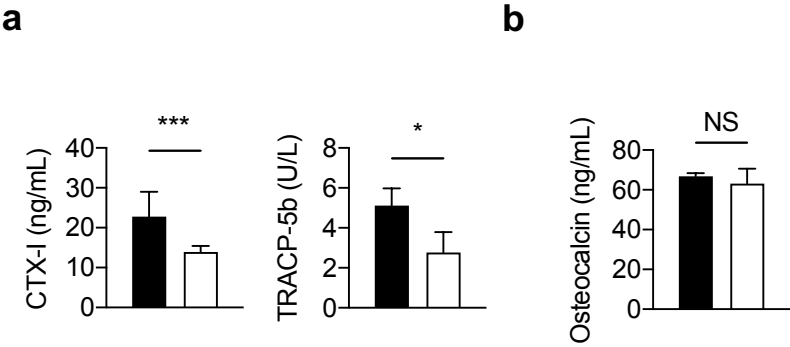

Supplement: Supplementary file 9 — Supplementary figure 7. Serum levels of bone resorption and formation markers [file 41413_2019_80_MOESM9_ESM.pdf]
