# Supplementary material for: IgSF11 regulates osteoclast differentiation through association with the scaffold protein PSD-95
Source: Bone Res. 2020 Feb 10;8:5. doi: 10.1038/s41413-019-0080-9 (PMC7010662; doi:10.1038/s41413-019-0080-9)
Supplement: Supplementary file 2 — Supplemental information legends [file 41413_2019_80_MOESM2_ESM.docx]

**Supplemental information legends**

**Supplementary figure 1. Cloning of IgSF11 as a novel gene involved in late-stage osteoclast differentiation**

(a) Multinucleation is a hallmark of late-stage osteoclast differentiation and bears some similarity to multinucleated giant cells (MGCs). We reasoned that osteoclasts and MGCs share molecular programs that govern multinucleation and that identification of genes that control osteoclast multinucleation could be useful to understand regulatory mechanisms of osteoclasts. We prepared tetraploid osteoclasts (OC4N) and tetraploid MGCs (MGC4N) as multinucleation-committed cells and monocytes (BMM2N) and performed comparative gene expression profiling. Because MGC fusion is dependent on STAT6-mediated IL-4 signaling, we also prepared *Stat6*^-/-^ monocytes (*Stat6*^-/-^BMM2N) and cells cultured with IL-3 + IL-4 (*Stat6*^-/-^MGC2N) to filter out potentially cell fusion-independent MGC-associated genes. We further filtered out genes that do not have a transmembrane domain, and as a result we identified the gene *Igsf11*. (b) Relative expression of IgSF11 in monocytes (BMM2N, *Stat6*^-/-^BMM2N, and *Stat6*^-/-^MGC2N) and multinucleation-committed cells (OC4N and MGC4N). Monocytes and multinucleation-committed cells were sorted and total RNAs were isolated. Results of the expression of IgSF11 are shown as mean ± SD of three independent experiments. (c) Q-PCR validation of IgSF11 expression in BMMs, osteoclasts and MGCs. Relative expression of IgSF11 compared to BMMs is shown. Data were normalized to 18S rRNA and are shown as mean ± S.D.

**Supplementary figure 2. Generation of IgSF11-deficient mice by CRISPR/Cas9 system**

(a) Diagram of the *Igsf11* exon 1 sgRNA. The gRNA sequence is underlined in black and the PAM sequence is in red. (b) Part of *Igsf11* exon 1 genomic sequences from IgSF11^+/+^ and F2 IgSF11^-/-^ mice. Start codon is shown in bold. (c) PCR genotyping of IgSF11^+/+^, IgSF11^+/-^ and IgSF11^-/-^ mice were shown. Genomic DNAs from mouse tails were amplified using primers described in (b). (d) Expression of IgSF11 protein from IgSF11^+/+^ and IgSF11^-/-^ mice. Whole cell lysates from mouse testis were used for immunoblotting with anti-IgSF11 antibody. Anti-Tubulin antibody was used as a control. (e) Growth curves for IgSF11^+/+^ and IgSF11^-/-^ male mice from 1 to 10 weeks old. Data are shown as mean ± S.D.

**Supplementary figure 3. IgSF11 deficiency does not affect osteoclast survival**

(a) Osteoclast survival assay. IgSF11^+/+^ and IgSF11^-/-^ BMMs were treated with M-CSF + RANKL for three days to induce osteoclasts. Then, cells were harvested and further cultured with RANKL in new plates for 24 hours. Cells were fixed and stained with TRAP. Live and dead cells were counted and frequency of live cells was shown. Asterisks indicate dead cells. (b) Activity of Caspase-3. IgSF11^+/+^ and IgSF11^-/-^ BMMs were treated with M-CSF + RANKL for three days to induce osteoclasts. Culture medium was changed with fresh medium with RANKL, and further cultured for 12 hours. Caspase-3 activity, which increases during apoptosis, was measured using caspase-3/CPP32 colorimetric assay. Data are shown as mean ± S.D. NS; not significant.

**Supplementary figure 4. NF-κB and ITAM-signaling in IgSF11-deficient cells**

IgSF11^+/+^ and IgSF11^-/-^ BMMs were stimulated with RANKL for the indicated times, and phosphorylation of IκB-α, Syk and PLCγ2 was examined by western blot using the indicated antibodies. Anti-actin antibody was used as a control.

**Supplementary figure 5. Expression of IgSF11 in bone cells *in vivo***

(a) Representative micrographs of bone sections of IgSF11^+/+^/TRAP-tdTomato/Col2.3-ECFP mice stained with anti-IgSF11 antibody. Left: A micrograph with TO-PRO-3 and anti-IgSF11. Middle: Same visual field as left with TO-PRO-3, anti-IgSF11, TRAP-tdTomato, and Col2.3-ECFP. Magnified views of yellow boxes numbered from i to iv were shown in right. (b) Bone sections of IgSF11^+/+^ and IgSF11^-/-^ mice were stained with anti-IgSF11 antibody. Scale bars represent 20 μm.

**Supplementary figure 6. Microcomputed tomography analysis of IgSF11^-/-^ female mice**

The femurs of 16 week-old IgSF11^+/+^ and IgSF11^-/-^ female mice were analyzed. Bone volume per tissue volume (BV/TV), trabecular thickness (Tb.Th), trabecular number (Tb.N), trabecular spacing (Tb.Sp), bone mineral density (BMD), and cortical thickness (Ct.Th) are shown. Scale bars represent 0.5 mm. Data are shown as mean ± S.D. *; p < 0.05, ***; p < 0.001, NS; not significant.

**Supplementary figure 7. Serum levels of bone resorption and formation markers**

Serum levels of (a) CTX-I and TRACP-5b, and (b) osteocalcin in 12 week-old IgSF11^+/+^ and IgSF11^-/-^ male mice were measured by ELISA. Data are shown as mean ± S.D. *; p < 0.05, ***; p < 0.001, NS; not significant.

**Supplementary figure 8. IgSF11 localizes to cell-cell contacts**

(a) Representative snapshots of wild-type BMMs retrovirally transduced EGFP-tagged IgSF11 followed by culture with M-CSF + RANKL. Fluorescent and bright field images were taken every 5 min (upper). Fluorescent signal intensity was shown in green and red (bottom). Scale bars represent 20 μm. (b) Expression of VISTA on BMMs and preosteoclasts. IgSF11^+/+^ and IgSF11^-/-^ BMMs and preosteoclasts (BMMs treated with RANKL for two days) were stained with APC-conjugated anti-mouse VISTA (MIH63) antibody. Results are representative of three independent experiments.
