# Supplementary material for: IgSF11 regulates osteoclast differentiation through association with the scaffold protein PSD-95
Source: Bone Res. 2020 Feb 10;8:5. doi: 10.1038/s41413-019-0080-9 (PMC7010662; doi:10.1038/s41413-019-0080-9)

Supplementary figure 1

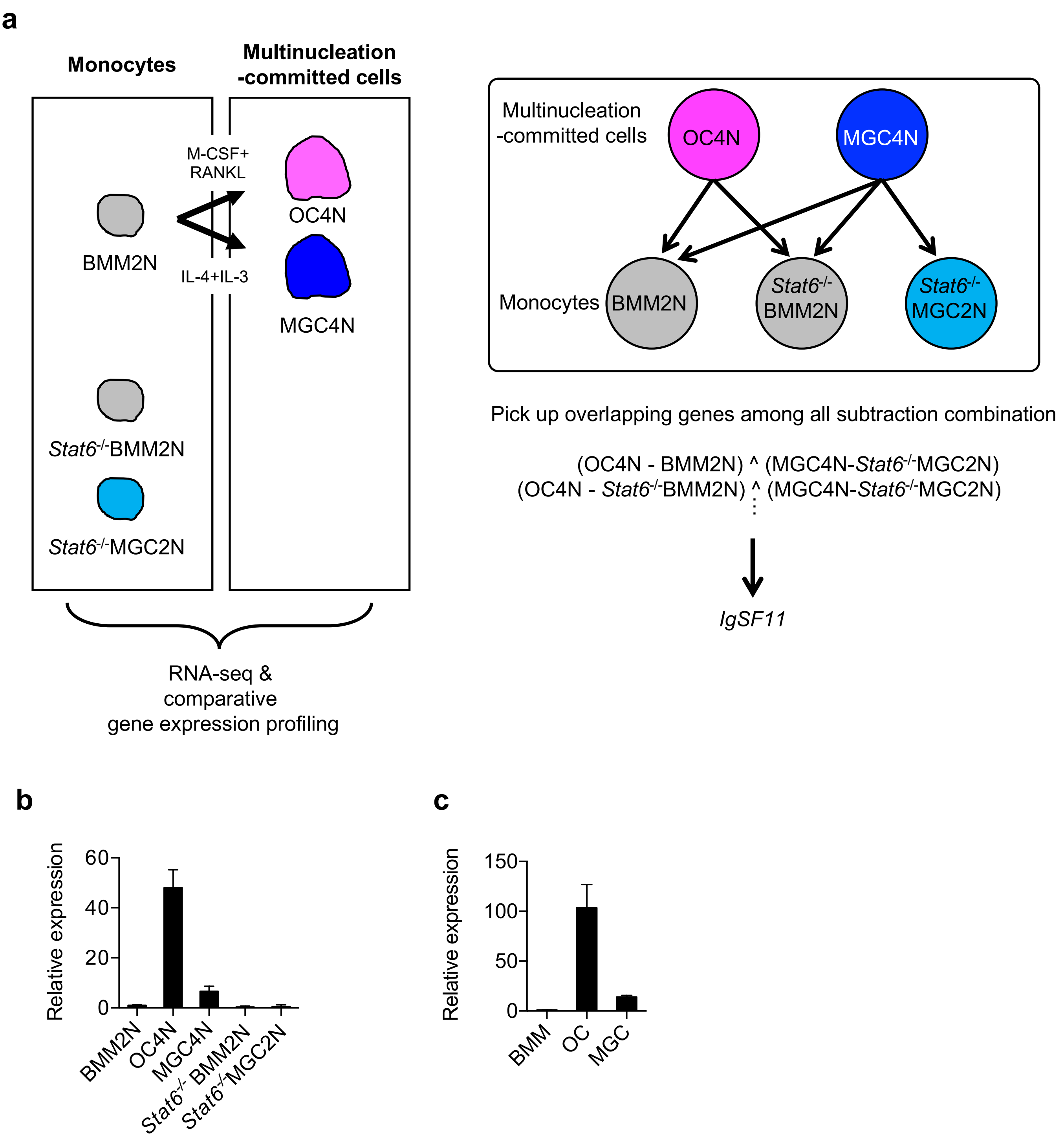

Supplement: Supplementary file 3 — Supplementary figure 1. Cloning of IgSF11 as a novel gene involved in late-stage osteoclast differentiation [file 41413_2019_80_MOESM3_ESM.pdf]
